# Environmental impact of computer-aided diagnosis in colonoscopy: a carbon footprint assessment of a prospective study cohort

**DOI:** 10.1055/a-2717-8365

**Published:** 2025-11-19

**Authors:** Olaolu Olabintan, Natalie Halvorsen, Robin Baddeley, Shin-ei Kudo, Ishita Barua, Masashi Misawa, Kensaku Mori, Claire Hunt, Jens Aksel Nilsen, Svein Oskar Frigstad, James E. East, Amit Rastogi, Cesare Hassan, Mette Kalager, Magnus Løberg, Amyn Haji, Øyvind Holme, Michael Bretthauer, Bu Hayee, Yuichi Mori, Shraddha Gulati

**Affiliations:** 1 King's Institute of Theraputic Endoscopy, King’s College Hospital NHS Foundation Trust, London, United Kingdom of Great Britain and Northern Ireland; 2 4616 Faculty of Life Sciences and Medicine, King's College London, London, United Kingdom of Great Britain and Northern Ireland; 3 6305 Clinical Effectiveness Research Group, University of Oslo, Oslo, Norway; 4 155272 Clinical Effectiveness Research Group, Oslo University Hospital, Oslo, Norway; 5 Wolfson Unit for Endoscopy, St Mark's Hospital and Academic Institute, London, United Kingdom of Great Britain and Northern Ireland; 6 King’s Institute of Therapeutic Endoscopy, King’s College Hospital NHS Foundation Trust, London, United Kingdom of Great Britain and Northern Ireland; 7 4615 Faculty of Natural Sciences, Imperial College London, London, United Kingdom of Great Britain and Northern Ireland; 8 220878 Digestive Disease Center, Showa University Northern Yokohama Hospital, Yokohama, Japan; 9 Graduate School of Informatics, Nagoya University, Nagoya, Japan; 10 Department of Medicine, Baerum Hospital, Vestre Viken Hospital Trust, Gjettum, Norway; 11 Translational Gastroenterology Unit, John Radcliffe Hospital, University of Oxford, Oxford, United Kingdom of Great Britain and Northern Ireland; 12 592235 Division of Gastroenterology and Hepatology, Mayo Clinic Healthcare, London, United Kingdom of Great Britain and Northern Ireland; 13 Oxford NIHR Biomedical Research Centre, Oxford, United Kingdom of Great Britain and Northern Ireland; 14 Division of Gastroenterology, University of Kansas Medical Center, Kansas City, United States; 15 437807 Department of Biomedical Sciences, Humanitas University, Pieve Emanuele, Italy; 16 Endoscopy Unit, IRCCS Humanitas Clinical and Research Center, Rozzano, Italy; 17 King's Institute of Therapeutic Endoscopy, King’s College Hospital NHS Foundation Trust, London, United Kingdom of Great Britain and Northern Ireland; 18 Faculty of Life Sciences and Medicine, King’s College London, London, United Kingdom of Great Britain and Northern Ireland; 19 Department of Medicine, Sørlandet Hospital Kristiansand, Kristiansand, Norway; 20 Clinical Effectiveness Research group, Oslo University Hospital, Oslo, Norway; 21 King's Institute of Therapeutic Endoscopy, King's College Hospital NHS Foundation Trust, London, United Kingdom of Great Britain and Northern Ireland

## Abstract

**Background:**

The environmental impact of computer-aided diagnosis (CADx) in colonoscopy is unknown. The potential to reduce greenhouse gas emissions by limiting unnecessary polypectomies may be offset by emissions associated with CADx devices. We quantified and compared the carbon footprint of colonoscopy with and without CADx.

**Methods:**

Life-cycle carbon emissions associated with endoscopic snares, polyp traps, histopathology, and CADx devices were estimated using life-cycle assessment software. The effect of CADx was estimated by the carbon dioxide equivalent (CO2e) emissions related to outcomes from a prospective cohort of patients who underwent colonoscopy with optical diagnosis under a leave-in-situ strategy (i.e. rectosigmoid polyps ≤5 mm predicted as non-neoplastic were not removed) with and without CADx assistance.

**Results:**

We analyzed 1134 individuals (median age 67 years, 59% male) with 1716 colorectal polyps. Colonoscopy with CADx reduced the number of snares and polyp traps from 567 to 543 (difference: 24) and histopathology submissions from 1343 to 1278 (difference: 65). However, the total carbon footprint increased from 832 kg CO2e (95%CI 815 to 847) without CADx to 946 kg CO2e (95%CI 927 to 965) with CADx, corresponding to an absolute increase of 114 kg CO2e (95%CI 80 to 150) or 0.10 kg CO2e per colonoscopy.

**Conclusion:**

Using CADx under a leave-in-situ strategy increased the environmental burden of colonoscopy.

## Abstract (de)

**Graphical Abstract:**

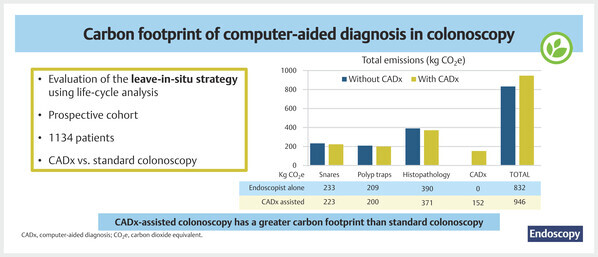

## Introduction

Technological advancements in medicine have improved patient care and outcomes but can increase greenhouse gas emissions 1 . Greenhouse gases absorb heat energy (the greenhouse effect), elevating the global surface temperature and potentially causing unwanted consequences such as drought, floods, wildfires, and reduction of air quality 2 . Greenhouse gases (such as carbon dioxide [CO _2_ ], methane, nitrous oxide) differ in their ability to trap heat. To compare them on a common scale, we report all emissions as CO _2_ equivalents (CO _2_ e), which is the mass of CO _2_ that would have the same 100‑year global warming potential as a given mixture of greenhouse gases. CO _2_ e enables standardized measurement and comparison of the climate impact of products and services using life-cycle analysis (LCA) 3 .

The health care sector has been estimated to account for 4.4% of the global greenhouse gas emissions, and gastrointestinal endoscopy has been shown to be an important producer of clinical waste 4 5 . Colonoscopies represent approximately 60% of endoscopy activity per year in the USA 6 , and considering that the estimated CO _2_ e for one colonoscopy is 55 kg, these procedures are an important field for enquiry 7 .

Colonoscopy is undergoing a major transformation through the integration of cutting-edge technologies such computer-aided diagnosis (CADx) of colorectal polyps. CADx may enhance endoscopists’ confidence and performance in optical diagnosis (prediction of polyp pathology), which could reduce unnecessary polypectomies and histopathological submissions 8 . However, the net environmental impact of this approach remains unknown; its potential to reduce the carbon footprint through avoided polypectomies may be offset by emissions associated with CADx devices, including machine learning processes, product development, and use.

The appraisal of medical innovations increasingly considers environmental impacts, alongside cost-effectiveness and clinical efficacy 9 . In the present study, we aimed to quantify the effect of CADx on the carbon footprint of the “leave-in-situ” optical diagnosis strategy used during colonoscopy.

## Methods

### Study overview

Our primary goal was to quantify the carbon footprint of optical diagnosis during colonoscopy with and without CADx under the leave-in-situ strategy, where rectosigmoid polyps ≤5 mm and predicted as non-neoplastic are not removed. To achieve this, we analyzed data from a prospective cohort study that measured the impact of CADx using an endocytoscope system (EndoBRAIN; Cybernet Systems Corp. [Ann Arbor, Michigan, USA] and Olympus Corp. [Tokyo, Japan]) during colonoscopy on endoscopists’ diagnostic performance and confidence in the optical diagnosis of diminutive rectosigmoid polyps 8 10 . A polyp would be assumed to be left in situ if the endoscopist had high confidence in the optical diagnosis with and without CADx.

The environmental impact of using CADx during a procedure was estimated by summarizing the CO _2_ e using LCA-derived carbon footprint data mapped to the individual patient-level outcomes from the cohort study. The primary analysis was comparison of the carbon footprint of colonoscopy with and without CADx.

This study was conducted in accordance with the Endoscopic Sustainability PrimAry Reporting Essentials (E-SPARE) guidelines developed by the European Society of Gastrointestinal Endoscopy (ESGE) to ensure transparent and standardized reporting of environmental impact in gastrointestinal endoscopy research 11 .

### Carbon footprint analysis

Life-cycle modeling was performed in SimaPro v9.5 (PRé Sustainability, Amersfoort, Netherlands). Material-specific inventory data for raw-material extraction, manufacturing, and transport came from Ecoinvent v3 (Ecoinvent Association, Zurich, Switzerland). We modeled the life-cycle of four components directly affected by optical diagnosis and CADx: snares, polyp traps, histopathological submissions, and the CADx system.

System boundaries covered cradle-to-gate plus use-phase emissions for every component: raw materials, manufacturing, packaging, shipping to the clinic, and in-procedure energy consumption. End-of-life disposal of the CADx unit was excluded owing to uncertain decommissioning pathways and long service life, and the clinical procedure itself (including scope, sedation, staff) was excluded because it was identical in both study arms.

Acquisition of primary data combined (i) direct measurements, (ii) manufacturer specifications (Cybernet Systems, Conmed Corporation), and (iii) data from prior LCAs 3 12 13 14 . Secondary data came from material-specific inventory data for raw material extraction, manufacturing, and transport sourced from the Ecoinvent v3 database, reflecting average global production conditions. We built the life-cycle inventory in SimaPro and characterized impacts with the ReCiPe 1.08 Midpoint (H) method.

### Items and assumptions

**Fig. 1:**
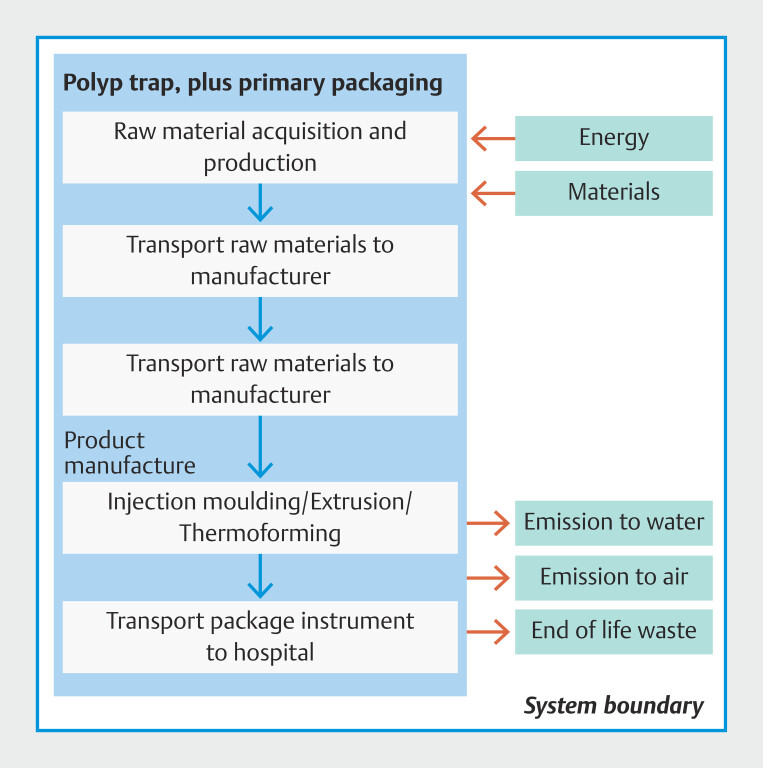
Polyp trap system boundary.

The items considered for inclusion in the analysis and related assumptions are shown in Table 1 . Each cold snare was assumed to contribute 0.41 kg CO _2_ e, derived from the average emission of an endoscopic snare established in a published LCA model 3 . Each polyp trap contributed 0.37 kg CO _2_ e based on a processed-based LCA of polyp traps. We included information about manufacturing, shipping, and disposal of the Optimiser multi-chamber polyp trap (ConMed Corporation, Largo, Florida, USA) ( Fig. 1 ; see also **Tables 1s–3s** in the online-only Supplementary Material). The model was informed by material composition and shipping route data provided by the manufacturer 15 .

**Table:** **Table 1** Summary of assumptions on the carbon dioxide emissions of the items affected by computer-aided diagnosis in colonoscopy and polypectomy.

Item	Emissions, kg CO _2_ e
Per snare	0.41
Per polyp trap	0.37
Per histopathology jar	0.29
CADx per procedure	0.31 ^1^ / 0.23 ^2^
CADx, computer-aided diagnosis; CO _2_ e, carbon dioxide equivalent. ^1^ In standby mode. ^2^ Unplugged.	

Histopathological submission was assumed to contribute 0.29 kg CO _2_ e per histopathology jar based on a previously published LCA on gastrointestinal biopsy material 13 . The processing of a diminutive polyp was confirmed by histopathologists working in the UK and Norway to be comparable to the processing of gastrointestinal biopsy material.

**Fig. 2:**
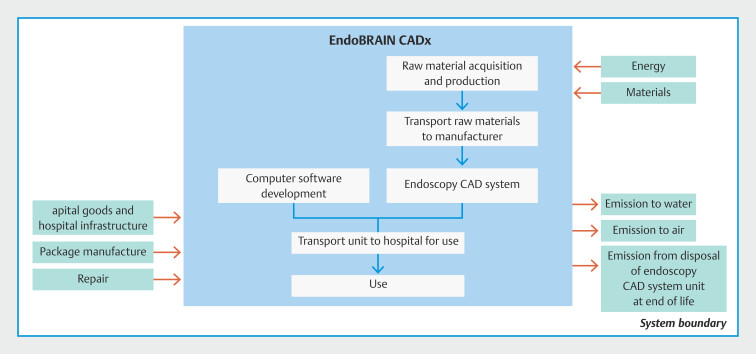
System boundary for computer-aided diagnosis (CADx: EndoBRAIN, Olympus, Tokyo, Japan).

We assumed that the CADx system device was in standby mode and produced 0.31 kg CO _2_ e per procedure based on a hybrid LCA including the development, manufacture, shipping, and use of the EndoBRAIN unit ( Fig. 2 , **Tables 4s–7s** ). Emissions generated during CADx software development were modeled by allocating most of the cost to a spend-based emission factor attached to the computer programming services sector in Ecoinvent. Disposal of the EndoBRAIN unit was not included in the analysis, given the long lifetime of the product and the as-yet undefined end-of-life handling of these units with potential for recycling. Assumptions about CADx activity were based on an assumed lifetime of 15 years with 15 000 procedures per lifetime, and the average time of colonoscopy and polypectomies with an active mode of 38 minutes during colonoscopy 16 17 18 . The energy consumption of the device varies depending on whether the device is in standby mode or unplugged when not used: 0.31 kg CO _2_ e or 0.23 kg CO _2_ e per procedure, respectively.

### Secondary analysis

We also conducted secondary analyses with emissions calculated for the following scenarios (see the **Supplementary Material** for detailed methodology).

1. Alternative polyp-handling strategies ( **Table 8s** ): a) total removal: resecting and submitting all polyps to histopathology; b) DISCARD-lite: all diminutive polyps in the right colon are presumed to be neoplastic, and the high-confidence non-neoplastic polyps in the rectosigmoid are left in situ 8 .
2. Number of CADx system uses according to a device lifetime of 1, 3, 15, and 20 years ( **Table 9s** ).
3. Follow-up colonoscopies required in the next 10 years ( **Table 10s** ).
4. Unplugging the CADx device outside of office hours ( **Table 11s** ).

### Statistical analysis

Statistical analyses were performed using R Commander v2.7–1 (R Foundation for Statistical Computing, Vienna, Austria) 19 . Data were treated as independent observations. Polyp-level data included the binary outcome of confidence in optical diagnosis (high or low) to estimate the number of histopathological submissions (each polyp), and patient-level data included the binary outcome of whether a polypectomy was performed (yes/no) to estimate the number of polyp traps, snares, and uses of the CADx device. The CIs for the proportion of each component were calculated by the binomial function test. We then converted count-based CIs into CO _2_ e CIs using first-order error propagation (the delta method). The two scenarios of diagnosis by endoscopist alone and CADx-assisted diagnosis were then compared and the CI for the difference was calculated.

### Study ethics and registration

The present study is part of the EndoBRAIN International trial, which has been approved by the ethical committee at each of the three participating centers and registered as a clinical trial (UMIN000027360). The present analysis was approved by the ethical committee of South-East Norway (no. C 605997).

## Results

### Patients

The database included 1242 individuals, of whom 37 did not meet criteria and 71 lacked registered information. We included data from 1134 individuals (median age 67 years; 59% male; 662 patients had 1716 polyps) who had undergone routine colonoscopy with CADx in Japan, Norway, and the UK between May 2019 and May 2021.

### Use of snare, polyp trap, and histopathological submissions

Among the 1134 patients, colonoscopies without CADx required 567 snares and polyp traps and 1343 histopathology submissions. In colonoscopies with CADx, 543 snares and polyp traps and 1278 histopathology submissions were required, resulting in 24 fewer snares and polyp traps, and 65 fewer histopathology submissions 8 .

### Carbon footprint without CADx

**Fig. 3:**
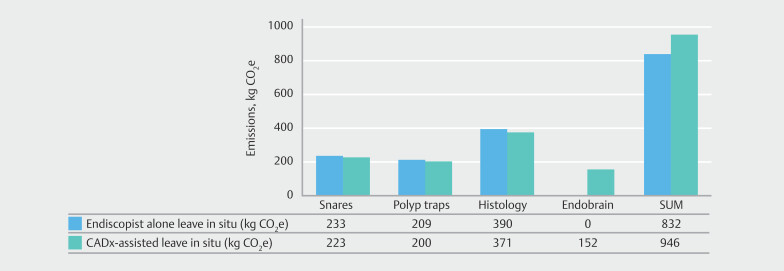
Estimated emissions (in kg CO _2_ e) of colonoscopy with endoscopist-alone and CADx-assisted leave-in-situ strategy. CADx, computer-aided diagnosis; CO _2_ e, carbon dioxide equivalent. EndoBRAIN, Olympus, Tokyo, Japan.

The total amount of CO _2_ e without CADx assistance was 832 kg (95%CI 815 to 847), or 0.73 kg per procedure ( Table 2 , Fig. 3 ). The contributory emissions were 233 kg CO _2_ e (95%CI 224 to 241) for snares, 209 kg CO _2_ e (95%CI 201 to 217) for polyp traps, and 390 kg CO _2_ e (95%CI 379 to 401) for the histopathological submissions ( Table 2 , Fig. 3 ).

**Table:** **Table 2** Estimated carbon dioxide emissions of colonoscopy when applying the leave-in-situ strategy with and without computer-aided diagnosis.

Emission variables	Emissions, kg CO _2_ e (95%CI)		
	Without CADx	With CADx	Difference (without–with CADx)
Snares	233 (224 to 241)	223 (214 to 230)	10 (–6 to 27)
Polyp traps	209 (201 to 217)	200 (192 to 207)	9 (–7 to 25)
Histopathology	390 (379 to 401)	371 (360 to 381)	19 (–2 to 41)
CADx	0	152 (145 to 167)	–152 (–167 to –145)
Total	832 (815 to 847)	946 (927 to 965)	–114 (–150 to –80)
CADx, computer-aided diagnosis; CO _2_ e, carbon dioxide equivalent.			

### Carbon footprint with CADx

The total amount of CO _2_ e with the CADx-assisted leave-in-situ strategy was 946 kg CO _2_ e (95%CI 927 to 965), or 0.83 kg per procedure ( Table 2 , Fig. 3 ). The contributory emissions were 223 kg CO _2_ e (95%CI 214 to 230) for snares, 200 kg CO _2_ e (95%CI 192–207) for polyp traps, 371 kg CO _2_ e (95%CI 360 to 381) for the histopathological submissions, and 152 kg CO _2_ e (95%CI 145 to 167) for the use of three CADx devices.

### Difference in carbon footprint between the two scenarios

Using CADx would result in an overall increase of 114 kg CO _2_ e (95%CI 80 to 150), or 0.10 kg CO _2_ e per procedure in the study cohort (n = 1134) ( Table 2 , Fig. 3 ). Use of CADx reduced emissions from snares, polyp traps , and histopathological submissions by 10 kg CO _2_ e (95%CI –6 to 27), 9 kg (95%CI –7 to 25), and 19 kg CO _2_ e (95%CI –2 to 41), respectively, but increased emissions by 152 kg (95%CI 145 to 167) due to the use of the CADx system.

### Secondary analysis

Alternative polyp-handling strategies were assessed ( **Table 8s** ). A strategy involving total polyp removal would produce 0.89 kg CO _2_ e in total per procedure. The DISCARD-lite strategy would produce 0.62 kg CO _2_ e in total per procedure.

Evaluation of different lifetime uses of the CADx system ( **Table 9s** ) showed that the 1, 3, 15, and 20 years scenarios for device lifetime would produce 2.76, 1.01, 0.309, and 0.265 kg CO _2_ e, respectively, from the CADx device alone per procedure.

There was no difference when comparing leave-in-situ with and without CADx (1219 colonoscopies) in terms of follow-up colonoscopies ( **Table 10s** ).

In colonoscopy with CADx and leave in-situ, unplugging the device outside of office hours would produce a total of 905 kg CO _2_ e (or 0.91 kg CO _2_ e per procedure), compared with 946 kg if keeping the machine in standby mode ( **Table 11s** ).

## Discussion

We investigated the effect of implementing CADx in colonoscopy procedures on carbon emissions. To our knowledge, our study represents the first attempt to quantify the carbon footprint of CADx by applying LCA data to individual patient-level outcomes from a prospective clinical study.

We primarily compared the total emissions of two approaches: optical diagnosis with and without CADx. Our analysis suggested a modest increase in carbon emissions when using CADx during colonoscopy. The use of CADx would slightly reduce the carbon footprint related to the requirement for snares, polyp traps, and histopathological submissions; however, the total carbon footprint when conducting colonoscopy without CADx would be 832 kg CO _2_ e, compared with 946 kg CO _2_ e with CADx assistance, equating to a 114 kg CO _2_ e (14%) increase in carbon emissions.

The 0.10 kg CO _2_ e increase per colonoscopy that we observed when CADx is used should be interpreted against fast-evolving decarbonization policies. In the UK, the Delivering a Net-Zero NHS roadmap aims to the be first net zero emissions health service in the world 20 . Our device-level LCA therefore provides essential evidence for assessing CADx under these requirements. Within gastroenterology, the ESGE Green Endoscopy Working Group urges endoscopy units to quantify and actively reduce procedural emissions. By following the E-SPARE checklist ( **Table 12s** ), our study offers a reproducible template for other AI-assisted innovations being considered under these society guidelines. In short, environmental performance will sit alongside diagnostic accuracy and cost-effectiveness in future health technology assessments. Mitigation options such as extended CADx device lifespans, routinely unplugging devices, or DISCARD-lite polyp-handling pathways could allow CADx to align more closely with net-zero trajectories while still delivering clinical benefit.

A strength of our study is that the assumptions regarding the effect of CADx on polyp management were derived from prospectively collected, real-world, observational data. The dataset included individual patient-level information about the effect of CADx on different populations including European and Asian patients, ensuring a robust set of data informing our calculations. These two strengths contribute to generalization of the study results.

Our study has several limitations. First, the application of environmental accounting methods to AI devices is poorly understood and relies on a number of assumptions. Second, the speed at which AI has entered clinical practice means that many of the calculations are also based on assumptions. Third, we did not include additional carbon emissions for disposal of CADx devices and follow-up after polypectomy in the primary analysis because we did not have observed data about these outcomes. However, sensitivity analyses showed there would probably be no difference in the total number of follow-up colonoscopies in 10 years. Finally, the CO _2_ e estimates in this study reflect the use of a CADx device manufactured in Japan. Manufacturing within Europe could reduce transportation-related emissions and lower the overall carbon footprint.

In conclusion, the use of CADx for leave-in-situ optical diagnosis may lead to a net increase in total emissions. As environmental accounting methods advance, more robust evaluations of the environmental impact of these technologies should be explored.

## Green Stamp Explained

The impact of endoscopy on the environment is well established, with colonoscopy, polyp removal, and histopathological assessment being important components. The use of artificial intelligence (AI) for establishing polyp diagnosis and implementing a leave-in-situ strategy was found to add a negative environmental impact to colonoscopy. Future strategies for implementing AI (and other technologies) should include an assessment of the environmental impact and measures of how to overcome it.

## Supplementary Material

Supplementary Material Zusatzmaterial
