# Supplementary material for: Environmental impact of computer-aided diagnosis in colonoscopy: a carbon footprint assessment of a prospective study cohort
Source: Endoscopy. 2025 Nov 19;58(3):303–9. doi: 10.1055/a-2717-8365 (PMC13077557; doi:10.1055/a-2717-8365)
Supplement: Supplementary file 1 — Supplementary Material [file 10-1055-a-2717-8365_27368887.pdf]

Supplementary material

Environmental impact of computer-aided diagnosis in colonoscopy: a carbon footprint assessment of a prospective study cohort

Olaolu Olabintan, Natalie Halvorsen, Robin Baddeley, Shin-ei Kudo, Ishita Barua, Masashi Misawa, Kensaku Mori, Claire Hunt, Jens Aksel Nilsen, Svein Oskar Frigstad, James E. East, Amit Rastogi, Cesare Hassan, Mette Kalager, Magnus Løberg, Amyn Haji, Øyvind Holme, Michael Bretthauer, Bu Hayee, Yuichi Mori, Shraddha Gulati

Endoscopy | DOI 10.1055/a-2717-8365 | © The Author(s).

Thieme

Table of Contents

Assessment of environmental impact 3

Life-Cycle-Assessment of a polyp trap 3

Unit analysis 3

Environmental lifecycle assessment 3

Table 1s Raw Material 4

Table 2s Transportation 4

Table 3s Waste handling 4

Life-Cycle-Assessment of EndoBRAIN - a hybrid analysis 5

Unit analysis 5

Environmental lifecycle assessment 5

Table 4s Product attributes 6

Table 5s Power requirements 6

Table 6s Transportation 7

Table 7s Electricity use 7

Secondary analyses 8

Table 8s Alternative scenarios 8

Table 9s EndoBRAIN sensitivity analysis according to lifetime uses 9

Table 10s Emission from surveillance colonoscopies 10

Table 11s Unplugged time consideration 10

Table 12s E-spare checklist 11

References 13

Endoscopy | DOI 10.1055/a-2717-8365 | © The Author(s).

Assessment of environmental impact

An LCA inventory of the relevant processes was compiled within SimaPro v9.5.0.0.[1] Lifecycle inventory emissions data were then characterised using the ReCiPe v1.1 Midpoint Hierarchist method (integrated within SimaPro).[2] The ReCiPe v1.1 Midpoint Hierarchist method evaluates 18 midpoint environmental impact categories, but for the purposes of our study this method was used to aggregate emissions and characterise into units of kilograms of carbon dioxide equivalents (kgCO_2e) to represent global warming impact, a ‘carbon footprint’.

Life-Cycle-Assessment of a polyp trap

Unit analysis

The total GHG emissions from all lifecycle stages were calculated with a 'cradle-to-grave' analysis to assess the environmental impact of the polyp trap. The Optimiser multi-chamber polyp trap (Conmed corporation, USA) was disassembled, and each distinct material component separated. The mass of each subcomponent was determined using a precision balance scale (Sartorious LP3200D). The raw material composition of each subcomponent and associated primary packaging was confirmed via personal correspondence with Conmed (**Table 1s**). The polyp trap was shipped overland to the UK via a heavy goods vehicle, and its disposal involved high temperature incineration (**Table 2s** and **Table 3s**). Material-specific data from Ecoinvent v3 was used for raw material extraction, manufacture, and transport.[3] Waste handling emissions were derived from a UK hospital study.[4]

Environmental lifecycle assessment

The carbon footprint of the manufacture and shipping of the polyp trap and its primary packaging is estimated at 362g CO_2e (**Fig. 2**). When emissions from waste handling are added, the lifecycle carbon footprint of the polyp trap and packaging is 368.2g CO_2e.

The biggest contributor to the impact (51.9%) is the production of polycarbonate, in keeping with its fraction of the product mass (51.5). Overland shipping of the product (3.45%) and end-of-life disposal (1.68%) were responsible for only a small fraction of the GHG emissions. Those processes contributing <4% to the impact are not displayed in the network diagram.

Endoscopy | DOI 10.1055/a-2717-8365 | © The Author(s).

Table 1s Raw Material

The raw material composition of each subcomponent and associated primary packaging of the Optimiser multi-chamber polyp trap (Conmed corporation, USA).

Component	Sub-component	Input	Weight (g)
Polyp trap	Cup and strainer	Polycarbonate	32.31
	Container Lid	High density polyethylene (HDPE)	5.55
	Connector	Methyl Methacrylate Butadiene Styrene (SBMMA)	2.13
	Tubing	Polyvinylchloride (PVC)	17.25
Packaging		Polyolefin (LDPE)	5.41
Total			62.65

G: grams

Table 2s Transportation

The overland shipping of the Optimiser multi-chamber polyp trap (Conmed corporation, USA) from Germany to the UK via a heavy goods vehicle.

Product	From	To	Mode of transport	Distance (km)
Polyp Trap and primary packaging	**Site of manufacture** *Gross-Gerau, Germany*	ATL Fulfillment, *Roemerberg, Germany*	Heavy goods vehicle	94
	ATL Fulfillment, *Roemerberg, Germany*	Conmed Brussels	Heavy goods vehicle	447
	Conmed, *Brussels*	Central UK city *Birmingham*	Heavy goods vehicle	568
	Manufacturer<br>DHL UK depot	Intermediate site<br>Receiving hospital	Courier	8<br>8
	Total Heavy Goods Vehicle			1109
	Total Heavy Courier			16

Km: kilometres; UK: United Kingdom. DHL and ATL Fulfilment are company names

Table 3s Waste handling

Waste handling emissions were derived from a UK hospital study.[5]

Component	Mass (g)	Mass (tonnes)	Disposal route	kgCO2e / tonne of waste	gCO2e
Polyp trap	57.24	0.00005724	High temperature incineration (clinical waste)	1074	6.14
Packaging (LDPE)	5.41	0.00000541	Low temperature incineration with energy from waste (Domestic waste)	172	0.09
**Total**					**6.23**

G: grams; kg: kilograms; CO2e: carbon dioxide emission; LDPE: low-density polyethylene

Endoscopy | DOI 10.1055/a-2717-8365 | © The Author(s).

Life-Cycle-Assessment of EndoBRAIN - a hybrid analysis

Unit analysis

The unit of analysis was defined as the lifetime use of an EndoBRAIN system (Cybernet Systems, Corp. Tokyo), a real-time artificial intelligence system used as a decision tool for polyp removal during colonoscopy.[6] Price, weight, expected lifetime uses, computer energy requirements for AI development for the EndoBRAIN unit and its subsequent use were obtained via the relevant technical specifications and direct communication with the manufacturers: Wincomm and Cybernet Corporation (**Table 4s** and **Table 5s**).

A 'cradle-to-gate' analysis was conducted, focusing on raw material extraction, hardware manufacture, software development (EndoBRAIN), and product transport to the hospital for use derived from the expected transport time of similar equipment available in Europe (EndoAID - CADe System OIP-1, Olympus). Due to the lack of specific databases, emissions for hardware manufacture were estimated using a typical desktop computer process. Capital goods, infrastructure, packaging manufacture, hardware repair, and end-of-life disposal were excluded. For EndoBRAIN software development, an environmentally extended input-output analysis was used based on economic expenditures. The Endo-AID was air-freighted from Japan to the UK, with additional road transport (**Table 6s**).

Electricity use during device operation was modelled for two scenarios. Scenario A: The EndoBRAIN is in active mode during its colonoscopy (38 mins). At all other times the device is considered ‘off’ but is never unplugged. Scenario B: The EndoBRAIN is in active mode during its colonoscopy (38mins). At all other times the device is considered ‘off’ but is unplugged outside of core endoscopy hours (9am – 5pm Mon-Fri). When unplugged, the EndoBRAIN requires no energy input. However, the unit draws 40.2W when off but not unplugged. Energy requirements were modelled on an annual basis and then calculated over the lifetime of the device and on a per procedure basis.

Environmental lifecycle assessment

The lifetime carbon footprint of the EndoBRAIN over 15 years, as modelled in this study is 4630 $kgCO_2e$ (Scenario A) or 3430 $kgCO_2e$ (Scenario B) (**Table 7s**). On a per procedure basis, assuming 15,000 procedures in the lifetime of an EndoBRAIN unit, this would equate to a procedural carbon footprint of 308gCO_2e (Scenario A) or 228gCO_2e (Scenario B). In scenario A, approximately 50% of the carbon footprint

Endoscopy | DOI 10.1055/a-2717-8365 | © The Author(s).

is attributable to the emissions generated during development of the software. This fraction increases when a more frugal approach to electricity use is adopted (scenario B).

Table 4s *Product attributes*

Price, weight, expected lifetime uses, computer energy requirements for AI development for the EndoBRAIN unit.

**EndoBRAIN**		
**Weight**		10kg
**Cost**		£38,625<br><br>($49031) *<br>*Deflated to 2002 = $28,987 ***<br>*Per case cost = $1.93 (28,987 / 15,000)*
**Number of cases uses per year**		1000 colonoscopies
**Lifetime case uses**		15,000***
**Mean time between failures**		3.12 years
**Average case length (colonoscopy using EndoBRAIN)**		38 minutes
**Energy requirements during use**	**Active**	191.3 Watts
	**Standby**	61.1 Watts
	**Off**	40.2 Watts

Kg: kilograms; £: British pound sterling; $: United states dollar.

** FT currency converter.*
*** CPI Inflation calculator May 2023 to May 2002.*
****Chip lifetime: 15 years.*

Table 5s *Power requirements*

Energy consumption metrics from the development and use of the EndoBRAIN unit.

**Power requirements for AI development**	
Computer processing time	113 hours
Energy consumption of computer	1200 Watts
Total computer electricity requirement for AI development	135.6 Kwh
Per Endobrain product *(assume develops 250 products)*	0.54 Kwh
Electricity requirement for AI development when allocated *per case (assume 15,000 cases per unit)*	0.000036 Kwh

Kwh: kilowatt-hour

Table 6s *Transportation*

EndoBRAIN is not commercially available in Europe. To estimate transportation related emission, we analysed product transport to the hospital for use derived from the expected transport time of similar equipment available in Europe (EndoAID - CADe System OIP-1, Olympus).

**ENDOAID Transport assumptions**					
**Product**	**Site of manufacture**	**Overseas travel mode**	**Overseas travel distance (km)**	**UK travel mode**	**UK travel distance (km)**
Olympus Endoscopy CAD System (ENDO-AID)	Japan	Courier<br><br>Heavy Goods vehicle<br><br>Air Freight	16<br><br>64<br><br>8190	Heavy Goods vehicle<br><br>Courier	64<br><br>16

Km: kilometre; UK: United Kingdom

Table 7s *Electricity use*

Electricity use during device operation was modelled for two scenarios. Scenario A: The EndoBRAIN is in active mode during its colonoscopy (38mins). At all other times the device is considered 'off' but is never unplugged. Scenario B: The EndoBRAIN is in active mode during its colonoscopy (38mins). At all other times the device is considered 'off' but is unplugged outside of core endoscopy hours (9am – 5pm Mon-Fri). When unplugged, the EndoBRAIN requires no energy input. However, the unit draws 40.2W when off but not unplugged.

**Procedural energy use**										
**Scenario**	**Active (191.3W)**		**Standby (61.1W)**		**Off (40.2W)**		**Unplugged (hours)**	**Annual (Kwh)**	**Total over 15 years (Kwh)**	**Total per procedure (Kwh)**
	Hours / year	Kwh	Hours / year	Kwh	Hours / year	Kwh				
**A**	633.3	121.2	0	0	8126	326.7	0	447.9	6718.5	0.44
**B**	633.3	121.2	0	0	1454	58.4	6672	179.7	2695.5	0.18

W: watt; Kwh: Kilowatt hour

Endoscopy | DOI 10.1055/a-2717-8365 | © The Author(s).

Secondary analyses

Table 8s *Alternative scenarios*

Total estimated kilograms of carbon dioxide emission of colonoscopy in CADx-assisted DISCARD-lite and Total removal. Total removal strategy is to remove and submitting all polyps to histopathology. This strategy excluded any optical diagnosis and would require 662 snares and polyp traps, and 1,716 histopathology submissions. [7] Total removal with resecting and submitting all polyps to histopathology would have a total CO_2e of 1013 kg (95% CI 968 kg to 1057 kg), or 0.89 kg CO_2e per procedure. The DISCARD-lite strategy is defined as all diminutive polyps in the right colon are presumed to be neoplastic, and only the diminutive polyps in the rectosigmoid evaluated as high-confidence are left in-situ.[8] The rest were submitted to histopathological evaluation. CADx-assisted DISCARD-lite gave a total CO_2e of 698 kg (95% CI 675 kg to 721 kg), or 0.62 kg CO_2e per procedure. This strategy would require 543 snares and polyp traps, and 424 histopathological submissions.[9]

**Emission variables**	**CADx-assisted DISCARD-lite, kg CO2e (95% CI)**	**Total removal: remove all polyps, kg CO2e (95% CI)**
Polyp traps	200 (192 to 207)	244 (233 to 268)
Snares	223 (213 to 245)	271 (259 to 298)
Histopathology	123 (117 to 135)	498 (475 to 547)
Endobrain	152 (145 to 167)	N/A
**SUM**	**698 (675 to 721)**	**1013 (967 to 1113)**

Kg: kilograms; CO2e: carbon dioxide emission; CADx: Computer-aided diagnosis; UK: United Kingdom.

Endoscopy | DOI 10.1055/a-2717-8365 | © The Author(s).

Thieme

Table 9s *EndoBRAIN sensitivity analysis according to lifetime uses*

The model was run with 4 x scenarios of CADx lifetime: The CADx system was simulated using different assumed lifetimes (1, 3, 15, and 20 years) for its use. The electricity demand over the product's lifetime was adjusted accordingly and dominates at longer lifetime. The emissions were allocated based on the number of uses during this period. The lifetime emissions would increase like 2,760 kg, 3,030 kg, 4,630 kg, and 5,300 kg CO_2e, whilst the per procedure emission would decrease to 2.76 kg, 1.01 kg, 0.31 kg, and 0.27 kg CO_2e.

	**Lifetime case uses**			
	1,000 (1 year)	3,000 (3 years)	15,000 (15 years)*	20,000 (20 years)
Production and shipping of hardware	same			
Software development	same			
Electricity required for software development (kWh)	0.54	0.54	0.54	0.54
Annual electricity demand (kWh)	447.9	447.9	447.9	447.9
Lifetime electricity demand (kWh)	447.9	1343.7	6718.5	8958
Total electricity demand	448.44	1344.24	6719.04	8958.54
Lifetime emissions (kg CO2e)	2760	3030	4630	5300
**Per procedure emissions (kg CO2e)**	**2.76**	**1.01**	**0.309**	**0.265**

*Main analysis

Endoscopy | DOI 10.1055/a-2717-8365 | © The Author(s).

Table 10s *Emission from surveillance colonoscopies*

Optical diagnosis might affect the total number of surveillance colonoscopies per individual for the next 10 years.[9] We analysed this potential effect by including the effect of CADx on the following simplified three-folded risk categories based on current guidelines.[10] We assumed 28.4 kg CO_2e per ordinary colonoscopy.[11]

1. No findings: individuals without adenomas have no surveillance colonoscopies in 10 years.
2. Low risk: individuals with ≤3 adenomas or ≤10 mm without tubule-villous characteristics or high-grade dysplasia has 1 surveillance colonoscopy in 10 years.
3. High risk: those that are not low risk or without findings have 2 surveillance colonoscopies in 10 years.

Optical diagnoses during colonoscopy without CADx had 26 false negative and 36 false positive polyps, with CADx there were 24 false negative and 52 false positive polyps. Both scenarios had a total of 1,219 surveillance colonoscopies in 10 years with estimated CO_2e of 34,620 kg.*

	Leave-in-situ		DISCARD lite	
Total removal (kg CO2e)	CADx (kg CO2e)	Endoscopist alone (kg CO2e)	CADx (kg CO2e)	Endoscopist alone (kg CO2e)
34676	34620	34620	37431	37403

Kg: kilograms; CO2e: carbon dioxide emission; CADx: Computer-aided diagnosis.

* The recommendations of follow-up colonoscopy vary on small details between regions which might affect surveillance colonoscopies in other ways than we have calculated. Our results of follow-up colonoscopies should therefore be validated by future studies on the effect of CADx on surveillance colonoscopies, which are currently lacking.

Table 11s *Unplugged time consideration*

Assuming the CADx systems were unplugged outside of core endoscopy hours (9am – 5pm Mon-Fri).

CO_2e of CADx per procedure (kg)	Total CO_2e in endoscopist alone leave in-situ	Total CO_2e: CADx-assisted leave in-situ
0.31kg[a]	832 kg (95% CI: 815 to 847)	946 kg (95% CI: 927 to 965)
0.23kg[b]	832 kg (95% CI: 815 to 847)	905 kg (95% CI: 886 to 924)

CO_2e: carbon dioxide equivalent emission; CADx: Computer-aided diagnosis; CI: Confidence interval; Kg: kilogram

a: in standby mode (main analysis)

b: unplugged when not used

Endoscopy | DOI 10.1055/a-2717-8365 | © The Author(s).

Table 12s *E-spare checklist*

Item	Recommendation	Reported on manuscript page
1. Title	Title should include the environmental impact and intervention, as appropriate.	1
2. Abstract	The abstract should include a description of the rationale, the intervention (if applicable), and the method used for environmental impact assessment.	3
3. Background/Motivation	Describe the scientific background and the rationale for the reported study.	4
4. Aims/Objectives	State the study hypothesis and objectives.	4
5. Impact on practice	Describe the potential impact of the study on GI endoscopy practice.	10
6. Study design (Goal & scope)	State and justify the goal and scope of the environmental impact assessment, defining: 6a the functional unit; 6b the boundary of analysis.	5-6
7. Key study parameters	Describe key study parameters, including: 7a clinical setting; 7b departmental characteristics; 7c time period/location; 7d multidisciplinary expertise.	4-5
8. Methodological approach	The methodological approach used to assess environmental impacts should be explicitly stated and justified.	5-6
9. Patient perspective	An evaluation of the patient perspective should be included if relevant to the study outcome measure(s).	n/a
10. Interventions	Describe any interventions performed, in sufficient detail to permit replication.	4-5
11. Environmental impacts defined	Define and justify the environmental impacts chosen for assessment, using standard terminology and units (e.g. kg CO_2e).	5
12. Assumptions/exclusions	Clearly state and justify any assumptions or exclusions.	5-7
13. Data sources/management	Data sources are reported based on the type of analysis applied (process- vs spend-based, primary vs secondary).	6
14. Resource allocation	Where resources are shared across activities, provide details on how they were assigned and justify the allocation method.	n/a
15. Bias	Clearly describe any attempts to address potential sources of bias (selection, measurement, confirmation).	10-11
16. Sample size	Provide an explanation as to how the sample size was calculated.	n/a
17. Variables handling	Describe how quantitative and qualitative variables were handled in the analyses.	7-8

Endoscopy | DOI 10.1055/a-2717-8365 | © The Author(s).

18. Statistical methods	Describe all statistical methods, including those to control confounders.	7-8
19. Subgroup/interaction analyses	Describe methods used to examine subgroups and interactions.	7
20. Missing data	Explain how missing data were addressed.	n/a
21. Emission factor origins	Data sources used for the impact assessment are described and justified (e.g. Ecoinvent, manufacturer specs).	5-6
22. Procedure characterization	Endoscopic procedures included in the analysis should be characterized (type/number, setting, sedation).	5
23. Device details	Details of the endoscopic devices used (type, brand, single-use vs reusable, recyclability) should be disclosed.	5 and supplementary material
24. GHG emissions by scope	The reporting of GHG emissions should include a breakdown according to Scope 1/2/3.	n/a
25. Outcome domains	Outcome data should be separated into pre-procedure, peri-procedure, and post-procedure domains.	5
26. Estimates & uncertainty	Disclose unadjusted and confounder-adjusted estimates with precision (e.g. 95% CI); explore sensitivity/uncertainty.	7-8
27. Main results	Describe the main results of the study according to the study objectives.	8-10
28. Social/financial implications	Discuss relevant social and financial implications of the findings (triple bottom line).	10-11
29. Generalizability	Discuss the generalizability and applicability of the results.	10-11
30. Limitations	Include a paragraph on the limitations of the study, including potential sources of bias and ways to overcome them.	11
31. Implications for change	If findings have clear implications for process, practice, or policy change, discuss next steps for stakeholders.	10-11
32. Conclusions & recommendations	Draw the main conclusions and recommendations for future study.	11

Endoscopy | DOI 10.1055/a-2717-8365 | © The Author(s).

References

1. PRé Sustainability. SimaPro. Version 9.5.0.0. Amersfoort, The Netherlands: PRé Sustainability; 2021.
2. Huijbregts M, Steinmann Z, Elshout P, et al. ReCiPe v1.1. Bilthoven, Netherlands: RIVM, Radboud University, CML, and PRé Consultants; 2016.
3. Ecoinvent Association. Ecoinvent v3. Zürich, Switzerland: Ecoinvent Association; 2023.
4. Rizan C, Bhutta MF, Reed M, Lillywhite R, et al. The carbon footprint of waste streams in a UK hospital. J Clean Prod 2021. doi:10.1016/j.jclepro.2020.125446.
5. Rizan C, Reed M, Bhutta MF, et al. Environmental impact of personal protective equipment distributed for use by health and social care services in England in the first six months of the COVID-19 pandemic. J R Soc Med 2021; 114: 250–263.
6. Cybernet Systems Corporation. EndoBRAIN. Tokyo, Japan: Cybernet Systems Corporation; 2019.
7. Rex DK, Kahi C, O'Brien M, et al. The American Society for Gastrointestinal Endoscopy PIVI (Preservation and Incorporation of Valuable Endoscopic Innovations) on real-time endoscopic assessment of the histology of diminutive colorectal polyps. Gastrointest Endosc 2011; 73: 419–422.
8. Atkinson NS, East JE, et al. Optical biopsy and sessile serrated polyps: Is DISCARD dead? Long live DISCARD-lite! Gastrointest Endosc 2015; 82: 118–121.
9. Halvorsen N, Barua I, Kudo SE, et al. Leaving colorectal polyps in situ with endocytoscopy assisted by computer-aided diagnosis: A cost-effectiveness study. Endoscopy 2025. doi:10.1055/a-2532-9282.
10. Hassan C, Antonelli G, Dumonceau JM, et al. Post-polypectomy colonoscopy surveillance: European Society of Gastrointestinal Endoscopy (ESGE) Guideline – Update 2020. Endoscopy 2020; 52: 687–700.
11. Lacroute J, Marcantoni J, Petitot S, et al. The carbon footprint of ambulatory gastrointestinal endoscopy. Endoscopy 2023; 55: 918–926.
